# Free-of-Acrylamide SDS-based Tissue Clearing (FASTClear) for three dimensional visualization of myocardial tissue

**DOI:** 10.1038/s41598-017-05406-w

**Published:** 2017-07-12

**Authors:** Filippo Perbellini, Alan K. L. Liu, Samuel A. Watson, Ifigeneia Bardi, Stephen M. Rothery, Cesare M. Terracciano

**Affiliations:** 1 National Heart and Lung Institute, Imperial Centre for Translational and Experimental Medicine, Imperial College London, Hammersmith Campus, Du Cane Road, London, W12 0NN UK; 2 Neuropathology Unit, Division of Brain Sciences, 4/F Burlington Danes Building, Imperial College London, Du Cane Road, London, UK; 3 Facility for Imaging by Light Microscopy, Sir Alexander Fleming Building, Imperial College London Exhibition Road, London, SW7 2AZ UK

## Abstract

Several pathologic conditions of the heart lead to cardiac structural remodelling. Given the high density and the opaque nature of the myocardium, deep three dimensional (3D) imaging is difficult to achieve and structural analysis of pathological myocardial structure is often limited to two dimensional images and of thin myocardial sections. Efficient methods to obtain optical clearing of the tissue for 3D visualisation are therefore needed. Here we describe a rapid, simple and versatile Free-of-Acrylamide SDS-based Tissue Clearing (FASTClear) protocol specifically designed for cardiac tissue. With this method 3D information regarding collagen content, collagen localization and distribution could be easily obtained across a whole 300 µm-thick myocardial slice. FASTClear does not induce structural or microstructural distortion and it can be combined with immunostaining to identify the micro- and macrovascular networks. In summary, we have obtained decolorized myocardial tissue suitable for high resolution 3D imaging, with implications for the study of complex cardiac tissue structure and its changes during pathology.

## Introduction

The complex, three dimensional (3D) arrangement of biological tissues is an essential determinant of their function and is often disrupted during disease. This is particularly important in the heart, where whole chamber, multicellular, cellular and sub-cellular structures play specific and highly regulated parts in cardiac performance. The ability to obtain high resolution 3D imaging is very valuable to visualise complex structures, such as extracellular matrix (ECM) or vasculature, which are difficult to investigate in two dimensional (2D) planes. In cardiac pathology the 3D representation of the vascular network or the fibrotic area of an infarcted region can provide crucial information for both basic and translational research. The conventional approach for 3D imaging involves sectioning whole tissue in ultrathin slices, imaging the slices and overlapping the consecutive images in registry to obtain a 3D reconstruction. This is a complex and laborious process which can easily generate out-of-registry and inaccurate results. A faster, less laborious and more accurate approach for 3D visualization would be to obtain images from deep planes directly, avoiding the sectioning of the sample altogether. However, light scattering through thick and opaque tissues, like the myocardium, and loss in excitation/emission efficiency result in low resolution and imaging depth, limiting the acquisition to only few µm depths from the surface of the samples for most microscopy techniques.

In the past few years important improvements in tissue clearing protocols have been made and new techniques such as CLARITY^1, 2^, 3DISCO^3^, iDISCO^4^, CUBIC^5^ have been developed. Optical clearing makes tissues transparent by matching the refractory index of different tissue layers. Light scattering is reduced and more light is able to travel across the sample for longer, allowing a much deeper penetration and a more defined 3D imaging. Optical clearing methodologies are still being optimized for simplicity, speed, cost-effectiveness and for the ability to be combined with immunolabelling of specific structures. In this study we used a Free-of-Acrylamide SDS-based Tissue Clearing (FASTClear) protocol, a combination of iDISCO technique with the omission of acrylamide-hydrogel in CLARITY^6^, for optical clearing of ventricular myocardium. FASTClear, as well as most of the other techniques, has been developed and used with brain preparations, but a specific protocol for adult cardiac tissue has not being reported. Here we describe for the first time a specific FASTClear protocol optimized for the 3D visualisation of adult myocardium which can be used with both freshly prepared or fixed samples from different species. This method dramatically reduces the time necessary to render the cardiac tissue optically transparent without damaging the ultrastructure; it is simple, robust, scalable, and inexpensive.

## Methods

### Myocardial tissue collection

Human left ventricular transmural biopsies were obtained from the explanted heart of four dilated cardiomyopathy end-stage heart failure patients undergoing cardiac transplantation. Human samples were provided by the NIHR Cardiovascular Biomedical Research Unit at the Royal Brompton and Harefield NHS Foundation Trust and Imperial College London. The study performed conforms to the principles outlined in the Declaration of Helsinki and the investigation was approved by a UK institutional ethic committee (Ref: 09/H0504/104 + 5 CBRU Biobank) and no organs/tissues were procured from prisoners. Informed consent was obtained from each patient involved in this study. All animal experiments complied with UK Home Office standard regulations as designated by the EU Directive 2010/63/EU. 2 male and 1 female 18 month old healthy Beagle dog hearts were kindly donated by GlaxoSmithKline, after the animals were necessarily sacrificed at the end of pharmacology/toxicology studies. Only control animals were used in this study. Canine hearts were removed following overdose of pentobarbital and confirmation of death. Both canine and human myocardial tissue blocks were quickly removed and immersed in 4 °C cardioplegia solution (Plegivex, Ivex Pharmaceuticals, UK; in mM: NaCl 147; KCl 16; MgCl2 16; CaCl2 1.2; Procaine hydrochloride), and transported to the laboratory (transport time approximately 2 hours).

### Myocardial slices preparation

Myocardial slices were prepared using transmural tissue blocks cut from the left ventricular free wall of human and canine hearts following the protocol described by Camelliti *et al*.^7^. The tissue block was glued (Histoacryl®, Braun, Germany) onto a thin layer of 4% agar and a high precision vibrating microtome (7000 smz, Campden Instruments Ltd., UK) was used to prepare viable myocardial slices. The slices were prepared in 4 °C oxygenated Tyrode Solution containing 30 mM BDM. The tissue was cut using a ceramic blade which was vibrating at 80 Hz with 2 mm amplitude and Z deflection < 1 μm. 300 μm slices were obtained with the blade advancing at 0.04 mm/s. Once cut the slices were kept in 4 °C oxygenated Tyrode Solution with BDM under mesh holders. FASTClear protocol can be used on freshly prepared slices as described below. Some slices were fixed with 4% paraformaldehyde (PFA) for 20 minutes at room temperature (RT) and stored in phosphate buffered saline (PBS) at 4 °C before being used for optical clearing.

### FASTClear on myocardial slices

**Figure 1:**
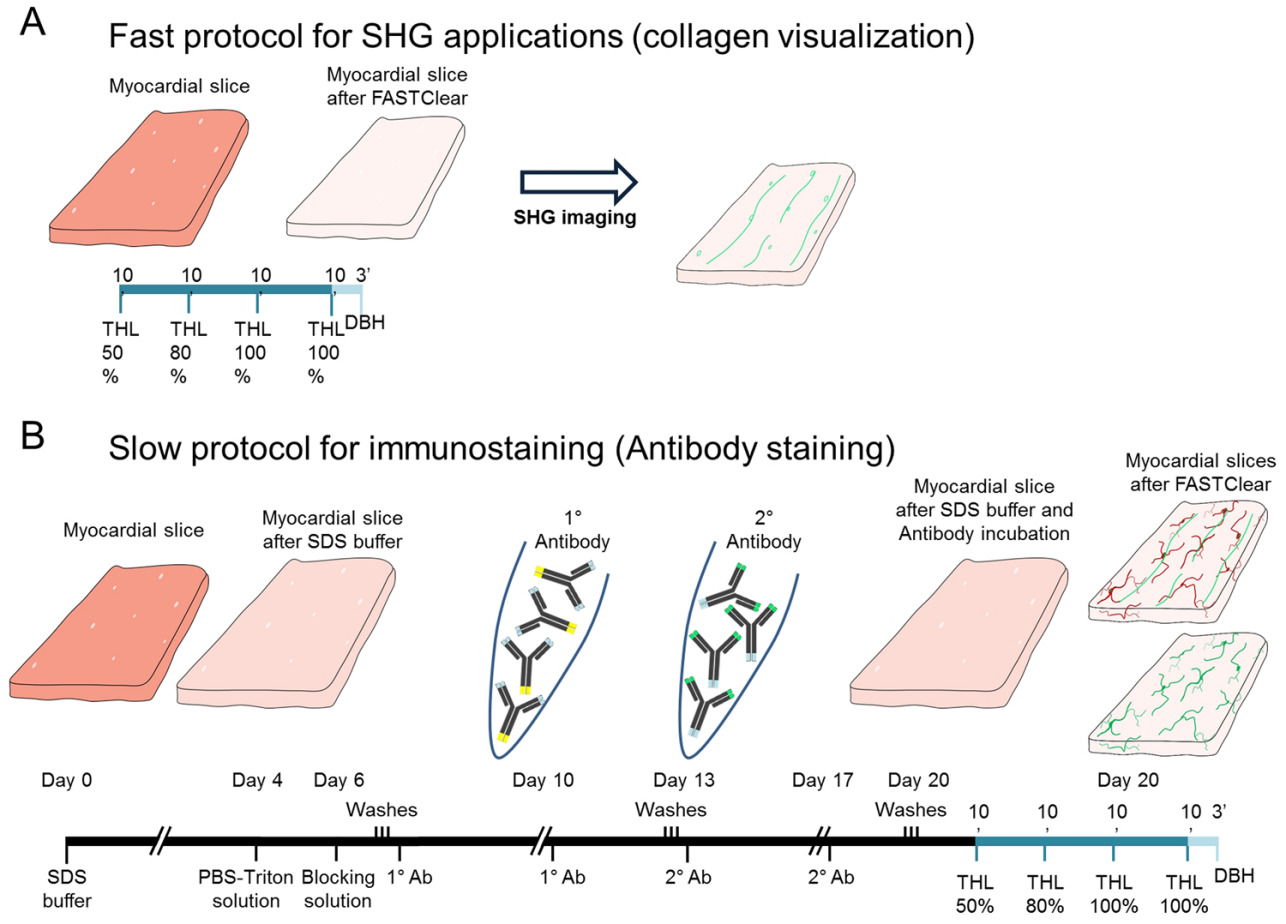
(**A**) Schematic representation of FASTClear protocol on freshly prepared or fixed myocardial slices and (**B**) combination of FASTClear protocol with immunostaining.

Starting from freshly prepared or fixed adult myocardial slices, tissue transparency was achieved in 45 minutes. A step by step protocol and the composition of the solutions are available in the Supplementary material. In brief, myocardial slices were immerse for 10 minutes in 50% tetrahydrofuran (THF), 80% THF, 100% THF, 100% THF and finally dibenzyl ether (DBE) until the tissue became optically transparent (approximately 3 minutes). A schematic representation of the process is illustrated on Fig. 1A. To combine tissue clearing with antibody staining the first step of the FASTClear protocol was to submerge fixed myocardial slices in 4% SDS buffer in the oven at 50 °C for 4 days to obtain delipidation of the tissue. We tested several SDS-buffer incubations (2 h, 8 h, 24 h, 2 and 4 days) and found that a 4 day incubation induced the best antibody penetration and labelling. During this period the samples partially lost their color and became almost optically transparent (opaque white). Next the samples were washed in PBS-Triton solution (1% Triton-X 100 in PBS) for 2 days on a rocker. Myocardial slices were blocked overnight at RT in 0.6 M glycine, 1% Triton X-100, 6% Donkey Serum, 20% dimethyl sulfoxide (DMSO) dissolved in PBS. The samples were then washed twice in PBS-Tween solution (for 1 hour, the primary antibody was diluted in the antibody-solution and the slices were incubated at RT for 1 week. After 4 days, more antibody was added to double the initial antibody concentration, directly in the solution, to each sample. Following three more washes in PBS-Tween solution the samples were left to wash for 24 hours on the rocker. Following the washes, the myocardial slices were incubated with the secondary antibody for 1 week on the rocker at RT. After 4 days, the solution was supplemented with additional secondary antibody to double the initial antibody concentration (Table 1). The samples were then washed 5 times in PBS-Tween solution (1 hour each) and left to wash ON. In case of double-staining after the initial staining for Vimentin the process was started again and Isolectin-B4 was added.

**Table 1:** Primary and secondary antibodies.

Primary antibody	Manufacturer	Initial concentration	Final concentration
Isolectin-B4	Life Technologies – I21414 (Biotin conjugated)	1:500	1:250
Vimentin	Abcam – ab92547 (Anti-Rabbit)	1:4000	1:2000
Secondary antibody	Manufacturer	Initial concentration	Final concentration
Alexa Fluor 568	Life Technologies – A11041 (Goat anti-Chicken)	1:2000	1:1000
Alexa Fluor 680	Life Technologies – A10043 (Donkey anti-Rabbit)	1:2000	1:1000

After SDS treatment and antibody incubation, the tissue was not completely transparent and therefore it was necessary to dehydrate the samples and match the refractive-index as per the 3DISCO protocol and described above. Myocardial slices were immerse for 10 minutes in 50% tetrahydrofuran (THF), 80% THF, 100% THF, 100% THF and finally dibenzyl ether (DBE) until the tissue became optically transparent. A schematic representation of the process is illustrated on Fig. 1B.

### Second harmonic generation imaging (SHG)

Tissues were mounted and visualised using a Zeiss LSM-780 confocal microscope or two-photon confocal microscope (Leica SP5). Collagen fibres were visualised using an adapted protocol previously described by Williams *et al*.^8^. SHG was performed on an upright Leica SP5 confocal microscope equipped with a Spectraphysics Mai Tai 690–1020 DeepSee multiphoton laser. The multiphoton was tuned to 850 nm and simultaneous dual imaging was performed for both Collagen I (SHG) detection at 410–430 nm and for cytoplasmic detail (autofluorescence) at 500–550 nm. Samples were mounted in dishes and Z-series of images were collected with dry and water dipping objectives. Images were acquired with a 10X and 25X magnification and processed with ImageJ (National Institutes of Health, USA) and Volocity (PerkinElmer Inc.).

### Quantification of ratio between the inter-capillary distance and the capillary diameter

In order to investigate whether sample shrinkage was homogeneous the ratio between the inter-capillary distance and the capillary diameter (before and after optical clearing) was measured. To evaluate the inter-capillary distance we measured the distance between capillaries in myocardial slices before and after FASTClear (Supp. Fig. 5, yellow lines), the average distance was respectively 21 ± 2.2 µm and 10 ± 1.0 µm (N = 6, 5 measurement/slice). Next we measured the capillary diameter which resulted being 5.36 ± 0.9 µm before and 2.87 ± 0.3 after FASTClear (N = 6, 5 measurement/slice, Supp. Fig. 5 green lines). 6 slices per conditions (originating from 3 dog hearts) were analysed and 5 capillaries and 5 inter-capillaries distance per slice were considered. The statistical comparison between groups was performed using a student-unpaired t-test in Prism5 software (GraphPad, San Diego, USA).

## Results

### FASTClear provides rapid and effective optical clearing of myocardial tissue and is compatible with homogeneous immunostaining

**Figure 2:**
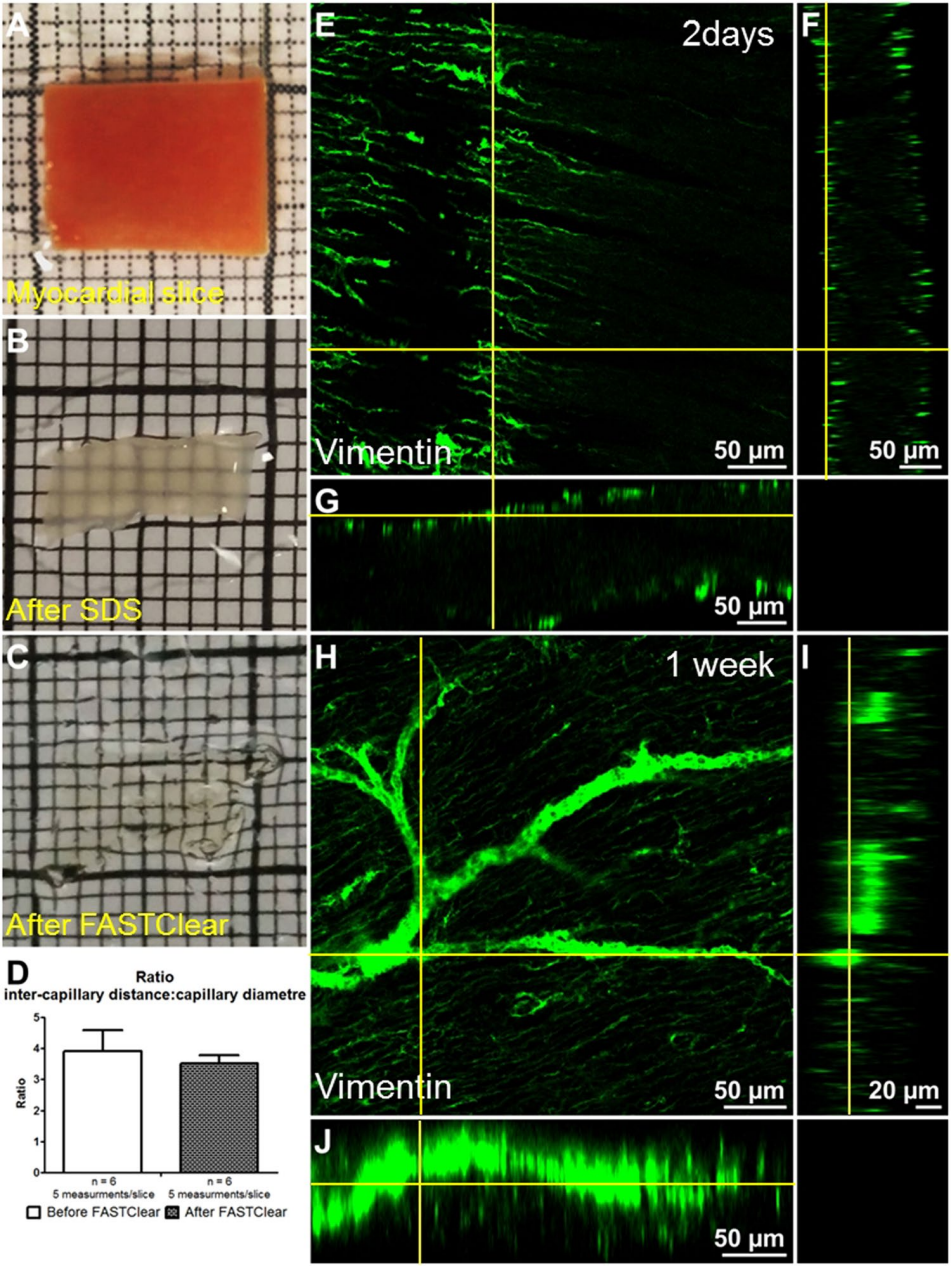
FASTClear provides an efficient myocardial tissue clearing protocol. (**A**–**C**) representative images of a myocardial slice before FASTClear, after SDS buffer treatment and after FASTClear protocol. (**D**) During tissue clearing the architecture and proportions of the myocardium is preserved and no changes in the inter-capillary distance to capillary diameter ratio were observed. Error bar = SEM. P > 0.05 T-test. (**E**–**G**) Orthogonal view of a myocardial slice after 48 hours antibody incubation, the labelling is limited to the surface of the preparation. (**H**–**J**) Orthogonal view of a myocardial slice after 1 week of antibody incubation. The labelling is homogeneously distributed and reached the core of the preparation. At least 3 slices were used for each experiment.

In this study we used 300 µm thick, freshly prepared or formalin-fixed adult human and canine myocardial slices. We have shown that freshly prepared viable myocardial slices have preserved structural and functional properties, they recapitulate the complexity of myocardial tissue, they are easy to handle and reproducible in origin and dimensions^7^. Here for the first time we show that using FASTClear (as adapted from iDISCO), which involve dehydration of tissue in series of increasing concentration of Tetrahydrofuran (THF) and refractive index matching with Dibenzyl ether (DBE), slices became transparent in less than one hour (Fig. 2A and C). We have also shown that fixation of the tissue is not required to achieve optical clearing and this further reduce the sample preparation by 20 minutes. It has previously been reported that with optical clearance methods the samples undergo tissue expansion or reduction^2, 6, 9^. Using the FASTClear method we observed a reduction in tissue size. This reduction is caused by the dehydration of the tissue and we observed that the magnitude of the shrinkage varied according to the state of the sample (fresh or fixed), the fixation process (4%PFA, methanol etc) or the length of storage (days, weeks or years). In order to investigate whether the shrinkage occurred homogeneously, affecting the cellular compartment and the ECM in a similar manner, the samples were stained for isolectinB4 (which labels capillaries) and the ratio between the inter-capillary distance and the capillary diameter was measured (6 slices prepared from 3 dog hearts, 5 measurement/slice, Fig. 2D). No statistical difference before and after optical clearing with FASTClear was observed, suggesting that this method homogeneously reduces the size of the different tissue components (Fig. 2D and Supp. Fig. 1), resulting in unchanged tissue structure.

An important aspect of 3D tissue imaging without sectioning is the ability to obtain homogeneous immunostaining, determined by the penetration of the antibodies throughout the whole thickness of the tissue^6^. 1 week of antibody incubation resulted in homogeneous distribution throughout the whole preparation (Fig. 2H–J). A shorter incubation period (48 hours) resulted in limited penetration mostly confined to the surface of the preparation (20–30 µm, Fig. 2E–G).

### FASTClear allows visualization of collagen throughout cardiac tissue slices using SHG microscopy

**Figure 3:**
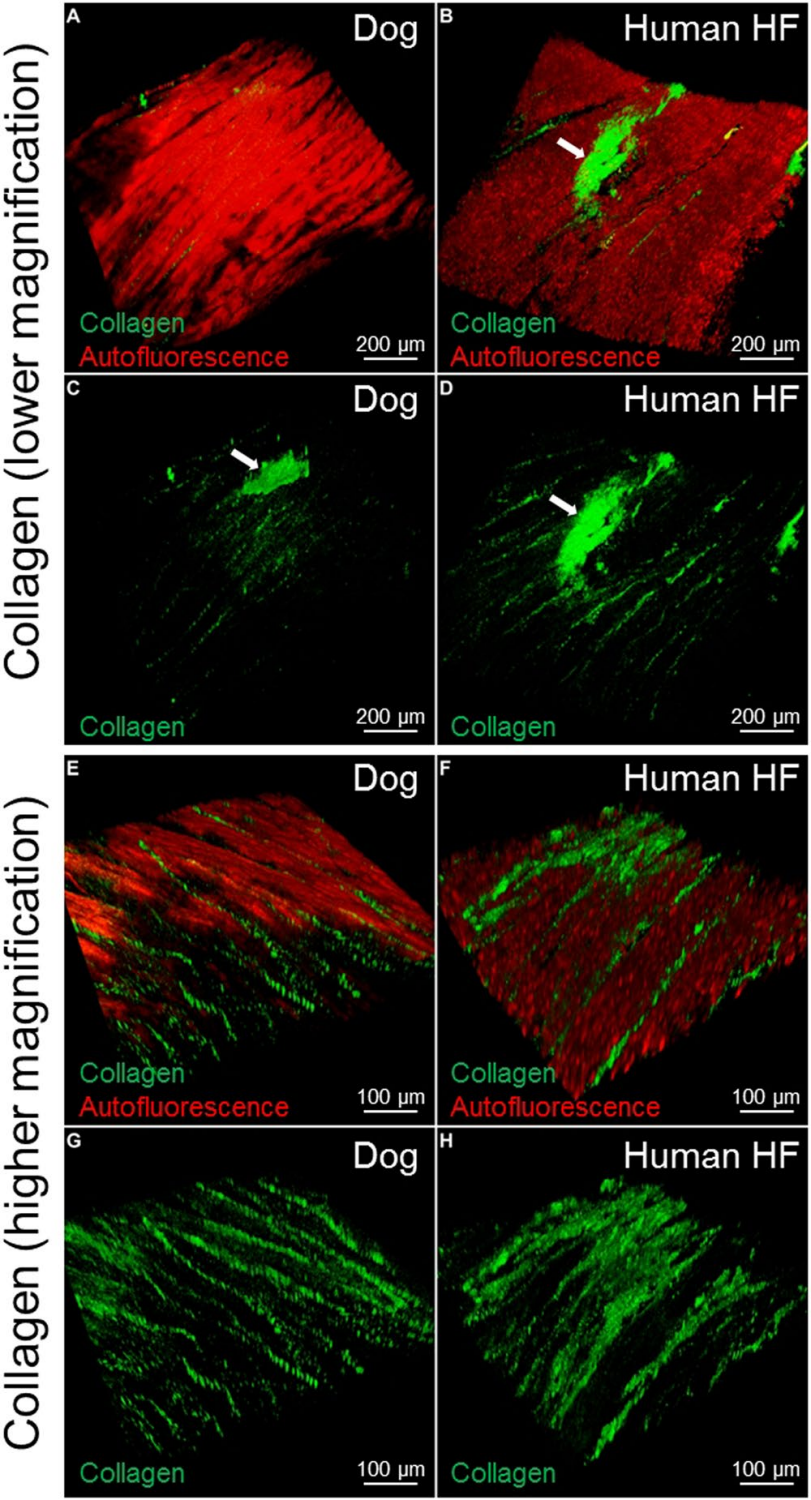
SHG imaging of Collagen type 1 in canine and human myocardial slices. Lower magnification of canine (**A**,**C**) and human (**B**,**D**) myocardial tissue. The autofluorescence (red) is used to show the structure of the myocardium; the collagen fibres (green) are located within the cardiac muscle fibres. The white arrows indicate the collagen in proximity to a large vessel. Higher magnification of healthy dog samples (**E**,**G**): the collagen is organised in strands between the muscle fibres; in human heart failure samples (**F**,**H**) the regular structure is partially lost and the collagen is less organised. At least 3 slices were used for each experiment.

In many cardiac diseases (infarction, heart failure etc), cardiac fibrosis is associated with worse clinical prognosis^10, 11^ and there is increasing interest in methods that permit a more detailed visualization, characterization and analysis of this phenomenon. Fibrosis results from increased collagen deposition due to activation of cardiac fibroblasts. SHG is currently used to visualise collagen fibres and therefore to study cardiac fibrosis^12, 13^. In brief SHG is a dye-free light emission arising from induced polarization rather than from absorption, with very limited photobleaching and phototoxicity commonly associated with fluorescence methods. This, in combination with a laser wavelengths in the NIR spectral range (700–1000 nm), achieves in soft or highly myelinated tissues high resolution images to a depth of several hundred microns which can be reconstructed into 3D representation of the analysed object^13, 14^. In denser tissues, such as the myocardium, the penetration of the light is significantly reduced to 50–80 µm^15^ and therefore optical clearing is required. The combination of SHG imaging with optical clearing in gastric tissue and skeletal muscle has further increased depth penetration by several fold^16, 17^. Here, after optical clearing was successfully achieved in healthy canine and human heart failing myocardial slices in approximately 45 minutes, we used SHG microscopy to image collagen fibres. Our data show that the signal is homogeneously distributed across the preparation and, in healthy myocardial slices, the collagen is organised in strands between the cardiac muscle fibres and in proximity to large vessels (Fig. 3A,C,E,F and Supp. Video 1). In the heart failure sample on the contrary, the collagen is more abundant and without regular structure (Fig. 3B,D,F,H and Supp. Video 2). These observations are in line with other studies^10, 11^ but here, for the first time, we were able to image and 3D reconstruct this changes in a large adult myocardial preparation which was ready for imaging in less than an hour. Myocardial slice collagen content and distribution could also be quantified and better characterized. Future experiments which include a larger number of samples and, possibly, a diversification into subgroups depending of the patient pathology, gender and age, are warranted.

### FASTClear allows immunostaining and confocal microscopy imaging of the micro- and macrovasculature in cardiac tissue

**Figure 4:**
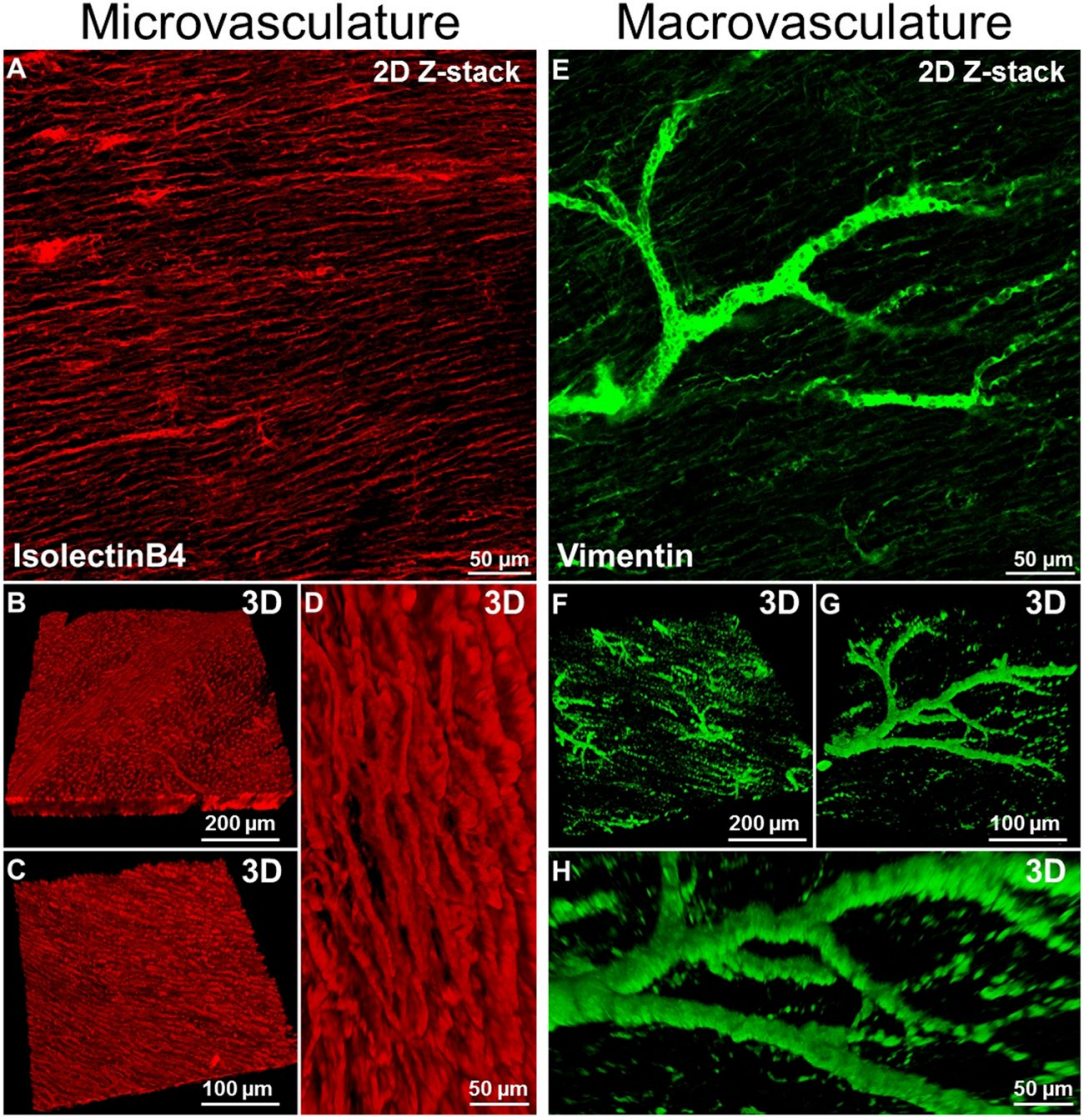
Immunostaining and imaging of micro and macrovasculature in cardiac tissue. (**A**) Two dimensional image of capillaries labelled with IsolectinB4. (**B**–**D**) Three dimensional reconstruction of myocardial tissue microvasculature at different magnifications. (**E**) Two dimensional image of larger vessels labelled with Vimentin. (**F**–**H**) Three dimensional reconstruction of myocardial tissue macrovascular network at different magnifications. At least 3 slices were used for each experiment.

Another important remodelling effect of cardiac disease is the modification of the vascular network^18, 19^. Immunostaining is one of the common and most informative methodologies used in research to visualise blood vessels. In order to obtain immunolabelling, however, it is required for the antigen to be preserved and available to bind the antibody. It has been reported that, during some tissue clearing protocols, damage to protein structure or protein loss might happen^2, 20, 21^. In a recent study Lai *et al*.^22^ showed no significant protein loss or visibly noticeable difference in immunostaining density after SDS clearing of formalin-fixed human brain tissue. In this study we used two different antibodies (isolectinB4 and vimentin) to identify the microvascular and the macrovascular network respectively in healthy dog myocardial slices. We showed that isolectinB4 stained the capillaries (Fig. 4A–D and Supp. Video 4) and 3D images could be reconstructed at different magnifications. High definition 3D reconstruction was also achieved using the vimentin antibody which labelled large blood vessels (Supp. videos 3 and 4) and provided a precise map of the macrovascular network.

**Figure 5:**
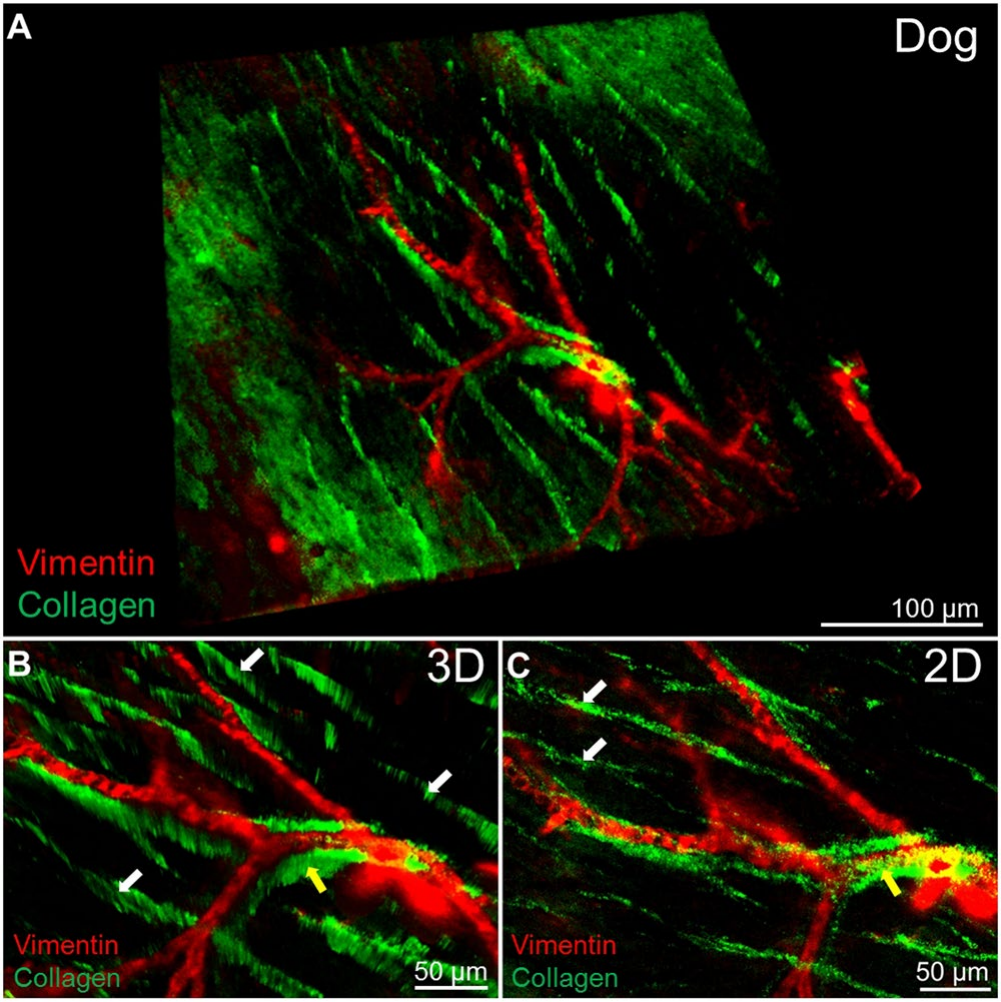
Combination of collagen type 1 imaging and macrovasculature staining. (**A**) Three dimensional reconstruction of the collagen (green) distribution in proximity to large vessels (vimenitin positive, red). (**B**,**C**) 3D and 2D higher magnification shows how the collagen separates myocardial muscle fibres (white arrows) but also surrounds larger vessels (yellow arrows). At least 3 slices were used for each experiment.

In order to further investigate the distribution of collagen in relationship with the cardiac vascular network we combined the images of the collagen network acquired with SGH microscopy with those of the blood vessels obtained with antibody staining and confocal microscopy. Figure 5A–C show high resolution and definition 2D and 3D imaging of the two structures simultaneously; the ability to obtain such images has important implications for studies of cardiac structural remodelling.

## Discussion

The high cellular density of myocardial tissue makes it difficult to image and, with the current imaging methods, the penetration is about 20–30 µm and 50–80 µm^15^ with confocal or with two photon microscopy respectively. The large majority of tissue clearing methods have been developed in whole body or intact brain samples and a protocol specific for myocardial tissue has not been described^1–6, 13, 23^. Tissue optical clearing has previously been observed in whole mouse heart as part of a protocol designed for whole body clearance and required 10 days to 2 weeks decoloration^23^. An improvement to the tissue clearing method for embryonic and postnatal cardiac tissue was achieved by Qureshi *et al*.^24^ with a shortening of the process to 5 days. In this study we set out to adapt and validate a FASTClear protocol for fast and simple 3D imaging of adult myocardial tissue. Our results demonstrate that: 1) tissue clearing can be achieved in human and canine myocardial tissue slices with a simple, rapid (45 minutes) and inexpensive method; 2) although during the process the tissue might undergo shrinkage, the overall tissue structure is preserved; 3) using SHG or confocal microscopy collagen distribution, the microvascular and macrovascular network can be clearly imaged in 3D at high definition throughout the whole preparation.

We used myocardial slices because they offer a standardised thickness of 300 µm while preserving the complex multicellular structure of the heart. We have shown that freshly prepared viable myocardial slices have preserved structural, biochemical and electrophysiological properties and they can be used for pharmacological studies^7^. Myocardial slices are currently very difficult to visualise in their entire volume with microscopy methods. After FASTClear treatment, the entire volume of myocardial slices achieved optical clearance in few minutes and the laser could easily travel across the preparation. As this method has already been applied to thicker brain samples (in the order of millimetres)^6^ we can speculate that it can be adapted to thicker cardiac preparations however further studies to prove this are warranted.

Depending on the optical clearing method, shrinkage or swelling during or at the end of the clearing procedure have been reported^2, 6^. In this study we observed a variable reduction in size depending on the state of the sample (fresh or fixed), the fixation process or the period of storage of the sample. Some attempts to reduce this undesired effect, mostly based on the combination of different existing protocols, have been described in literature but with limited success^25^. To investigate whether the shrinkage we observed with FASTClear modifies specific cellular structures, visualising the cell membrane directly would be desirable. However, during tissue clearance most of the lipid membranes are removed 1 Lipid membranes provide structural integrity and retain biomolecules. Removing these membranes would induce loss of some cellular and molecular information and this explain why several membrane-bound receptor staining don’t work. However the tissue ultrastructure and cellular morphology is preserved and many other information can be acquired. Because the visualisation of capillaries resulted very reliable with FASTClear, we opted to determine whether the capillary diameter and the distance between capillaries are reduced by a similar or a different amount. In line with the results published from Chung *et al*.^20^, our data showed no differences before and after FASTClear in the ratio of these two measurements. A preserved tissue ultrastructure, surface and cellular morphology have also been observed by Lai *et al*.^22^ in mouse brain tissue treated with FASTClear protocol. These authors applied FASTClear to mouse brain tissue, and using a scanning electron microscopy showed that this method does not significantly alter tissue ultrastructure and cellular morphology. Taken together these observations indicate that, despite shrinkage, the spatial relationship between tissue structures is maintained.

The FASTClear protocol combines the SDS-buffer delipidation step from CLARITY with the THF/DBE clearing step from iDISCO. Although it is not necessary to achieve transparency, SDS is a protein denaturant and, as a result, it induces myocardial tissue decoloration (the sample became opaque white) and has the additional effect of antigen retrieval which improves immunolabelling^6^. Following paraformaldehyde fixation or tissue clearing the antigens can be damaged or hidden and therefore antigen retrieval is often needed. Vimentin, for instance, is commonly used as a marker for cardiac fibroblasts which are located in the interstitial space between cardiomyocytes and in proximity to blood vessels. When FASTClear protocol was applied to myocardial slices, the antigens changed and the signal was limited to the large vessels. For this reason we recommend the Vimentin antibody, specifically after FASTClear tissue clearing, as a reliable marker to identify the macrovascular network.

After the SDS treatment the samples were incubated with antibodies for immunostaining for several days. 48 hour incubation was not sufficient to achieve a homogeneous distribution of the antibody in the preparation but this was achieved with 1 week incubation. Ongoing studies on human brain tissues using this technique has suggested prolonged SDS delipidation will further improve immunostaining and subsequent clearing (unpublished data). A method to further improve antibody penetration was to progressively increase the antibody concentration during the incubation period. Vascularization and/or the structural biochemical properties of the tissue have been proposed to induce heterogeneity in the antibody penetration in brain and spinal cord samples^6^. In our study using myocardial slices, this phenomenon was not observed and the antibody was homogeneously distributed throughout the sample.

Many cardiac diseases are associated with interstitial fibrosis, a process characterized by excessive deposition of collagen, in particular collagen type I^26, 27^. SHG has been used to visualise these structural alterations in cardiac tissue^15^. In line with the observations from Campagnola *et al*.^13^, optical clearing of myocardial slice significantly improved the laser penetration and the imaging resolution. The advantage here is that for collagen visualization and quantification, the tissue clearing process and the SHG imaging require only few hours compared to the conventional histology methods (days). Three-dimensional collagen analysis of laboratory or clinical specimens will improve the ability to distinguish pathological lesions and as a consequence improve the sensitivity and objectivity of diagnosis and treatments. Further improvements to this method could be achieved using higher-power objectives, longer working distance lenses and the development of tissue clearing protocols. SWITCH protocol, for instance, seems to facilitates antibody penetration with the help of glutaraldehyde in fixation (instead of paraformaldehyde) and SDS in antibody solution to achieve deep penetration of antibodies^28^. The development of single-chain antibody, nanobodies and aptamers^29^, which are able to penetrate in depth in the tissue and specifically bind biological markers, will lead to further enhancement in the imaging and increase the 3D resolution.

In summary, FASTClear is a greatly simplified and user-friendly tissue clearing protocol and here we specifically optimized it for adult cardiac tissue samples. This methodology will be a crucial tool for 3D investigation of the cellular and whole tissue organization of collagen and vascular network and provide a more integrated and comprehensive understanding of function and structure of cardiovascular tissue in health and disease.

## Electronic supplementary material

Supp video 1

Supp video 2

Supp video 3

Supp video 4

Supplementary material
