# Supplementary material for: Free-of-Acrylamide SDS-based Tissue Clearing (FASTClear) for three dimensional visualization of myocardial tissue
Source: Sci Rep. 2017 Jul 12;7:5188. doi: 10.1038/s41598-017-05406-w (PMC5507863; doi:10.1038/s41598-017-05406-w)
Supplement: Supplementary file 5 — Supplementary material [file 41598_2017_5406_MOESM5_ESM.pdf]

(SREP-17-11391A)

Supplementary Data

Free-of-Acrylamide SDS-based Tissue Clearing (FASTClear) for three dimensional visualization of myocardial tissue

Filippo Perbellini, Alan K L Liu, Samuel A Watson, Ifigeneia Bardi, Stephen M Rothery, Cesare M Terracciano*

Video 1. Three dimensional reconstruction of type 1 collagen localization in healthy canine cardiac tissue. Vimentin (green) labelled the macrovasculature and the autofluorescence (red) was used to show the structure of the tissue. The collagen is nicely organised in stripes which separate the muscle fibres.

Video 2. Three dimensional reconstruction of type 1 collagen localization in human heart failure cardiac tissue. Vimentin (green) labelled the macrovasculature and the autofluorescence (red) was used to show the structure of the tissue. In heart failure tissue the collagen organization is partially lost.

Video 3. Three dimensional reconstruction of myocardial tissue macrovasculature. Vimentin (green) labelled the macrovasculature and the autofluorescence (red) was used to show the structure of the tissue.

Video 4. Three dimensional reconstruction with a higher magnification of myocardial tissue macrovasculature. Vimentin labelled the macrovasculature (green) and isolectinB4 (red) the microvasculature.

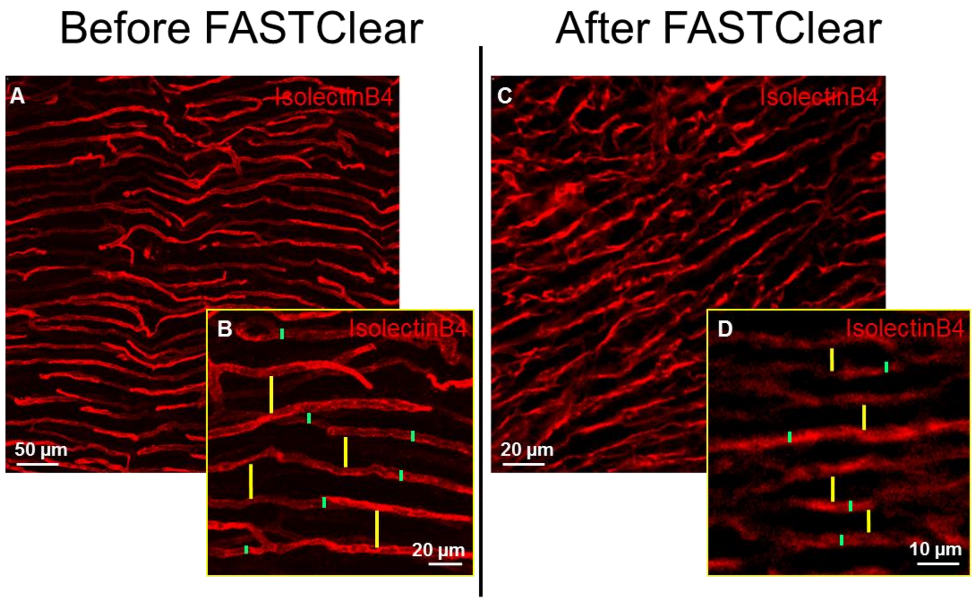

Supp Fig 1. Measurement of inter-capillary distance and capillary diameter . 6 dog slices were labelled with IsolectinB4 before and after FASTCLear. The inter-capillary distance (yellow lines) and the capillary diameter (green lines) were measured. 6 slices originating from 3 dogs were used and 5 measurements per slice were considered. After

tissue clearing the samples reduced in size therefore a higher magnification, as shown by the scale bars, was required for imaging the capillaries.

Supplementary material: Step by step protocol

FASTClear protocol

1. **Immersion in refractive index matching medium (freshly prepared or 4% PFA fixed myocardial slices)**

- <u>Caution: perform this step in the fume hood</u>
- 50% tetrahydrofuran (THF) 10 minutes at Room Temperature (RT)
- 80% THF for 10 minutes at RT
- 100% THF for 10 minutes at RT
- 100% THF for 10 minutes at RT
- dibenzyl ether (DBE) at RT until the tissue become optically transparent (approximately 3 minutes).

FASTClear and immunolabelling of myocardial slices

1. Fixation in 4% paraformaldehyde (PFA) 20 minutes @ room temperature

2. Immerse in 4% SDS buffer solution 4 days @50ºC oven

- This step improves antibody labelling. The samples partially lose their color and became almost optically transparent (opaque white).
- Daily change of 4% SDS buffer buffer can further improve antibody labelling.

4. Wash in 0.2% PBS-Triton solution 2 x 1 day @ RT on the rocker

5. Blocking and permeabilization in blocking medium Overnight @ 37ºC

6. Wash in 0.2% PBS-Tween solution 2 x 1 hr @ RT on the rocker

7. Primary antibody incubation 7 days @ 37ºC

- After 4 days, add more antibody in order to double the initial antibody concentration Optimal concentration and days of incubation vary between antibodies.
- If multiple antigen labelling is required, it is recommended to perform immunolabelling sequentially. However we also combined multiple antibodies in the same primary antibody solution and obtained good signal and deep antibody penetration.

8. Wash in 0.2% PBS-Tween solution3 x 1 hr @ RT on the rocker; then overnight @ 37ºC

9. Secondary antibody incubation 7 days @ 37ºC

- Same as Step 7 above

10. Wash in 0.2% PBS-Tween solution5 x 1 hr @ RT on the rocker; then overnight @ 37ºC

11. Immersion in refractive index matching medium RT until transparency is reached

- Caution: perform this step in the fume hood
- 50% tetrahydrofuran (THF) 10 minutes at RT
- 80% THF for 10 minutes at RT
- 100% THF for 10 minutes at RT
- 100% THF for 10 minutes at RT
- dibenzyl ether (DBE) at RT until the tissue become optically transparent (approximately 3 minutes)

Reagents

4% SDS buffer solution

- 12.366g Boric acid
- 40g SDS
- 1L distilled H_2O
- pH to 8.5 with NaOH

0.2% PBS-Triton solution

- 2ml Triton-X 100 in 1L 1x PBS
- Add 1g (0.1% wt/vol) sodium azide

0.2% PBS-Tween solution

- 2ml Tween-20 in 1L 1x PBS
- Add 1g (0.1% wt/vol) sodium azide

Blocking medium (100ml)

[0.6M glycine, 0.2% Triton X-100, 6% Donkey Serum, 20% DMSO, 0.01% sodium azide in 1x PBS]

- 2.252g glycine
- 200ul Triton X-100
- 6ml donkey serum
- 20ml DMSO
- 0.005g sodium azide
- 10ml 10xPBS
- Add water to 100ml

Antibody diluent (100ml)

[0.2% Tween-20, 5% DMSO, 3% Donkey serum, 0.01% sodium azide in PBS]

- 0.2ml Tween-20
- 5ml DMSO
- 3ml Donkey serum
- 0.01g sodium azide
- 10ml 10xPBS

- Add water to 100ml
